# In vitro and in vivo hemocompatibility study of fish swim bladder-derived biomaterials for the application of chronic venous disease

**DOI:** 10.1007/s10856-025-06969-1

**Published:** 2025-12-17

**Authors:** Qiushuo Zong, Yunfei Chen, Yuanyuan Kong, Zhihong Wang, Yiping Dang, Jing Liu

**Affiliations:** 1 Tianjin Key Laboratory of Biomaterial Research, Institute of Biomedical Engineering, Chinese Academy of Medical Sciences and Peking Union Medical College, Tianjin, China; 2 Department of Vascular Surgery, Union Hospital, Tongji Medical College, Huazhong University of Science and Technology, Wuhan, China; 3 Department of Cardiovascular Surgery, Second Affiliated Hospital of Kunming Medical University, Kunming, China; 4 Institute of Transplant Medicine, Nankai University School of Medicine, Tianjin, China

## Abstract

Chronic venous disease (CVD) is a prevalent condition affecting the venous system of the lower limbs, characterized by venous hypertension and regurgitation resulting from congenital or acquired venous valve insufficiency. The global morbidity of CVD is as high as 69.94%. Prosthetic venous valve replacement provides a new therapeutic option. However, to mimic natural venous valves, the leaflets of prosthetic venous valves need to be as thin as ~200 μm, and better resistance to thrombosis is required due to the physiological characteristics of the venous system. Identifying suitable materials for the venous system is critical for the creation of prosthetic venous valves. Fish swim bladders have proper mechanical strength, durability and biocompatibility, making them one of the possible biomaterials for application in CVD. For the blood contact safety in the application of chronic venous disease, in vitro and in vivo studies were used to evaluate the blood compatibility, as specified in ISO 10993-4. The results showed that the swim bladder was comparable to the commercially available ePTFE material in the venous system and has the potential to be a raw material source for the development of medical devices for CVD.

**Graphical Abstract:**

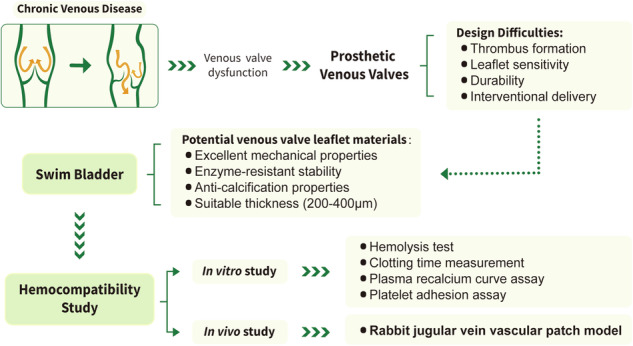

## Introduction

Chronic venous disease (CVD) is a disease of the venous system of the lower extremities caused by venous hypertension and venous regurgitation, with venous valve insufficiency accounting for 70%-80% of its causes. The annual incidence rate of CVD is ~2.3% and is increasing with the ageing of society. The current morbidity of CVD is 69.94% worldwide [1, 2]. Clinical symptoms usually include edema, lower extremity soreness or weakness, varicose veins, and even ulcers [3, 4]. Advanced age, being overweight, a history of pregnancy or lower extremity venous thrombosis, a family history of CVD, long-standing occupations, smoking, and lack of daily exercise are common risk factors for CVD [5]. In patients with severe deep venous regurgitation, conservative treatments represented by compression, medications, and superficial vein closure surgery have limited efficacy because they do not restore the function of the venous valves from the underlying etiology [6]. Valvuloplasty and surgery to reduce the venous diameter are currently the mainstays of venous regurgitation in clinical practice. Both endovascular and extravascular valvuloplasty are associated with gradual reoccurrence of occlusion or regurgitation after surgery [7, 8], while the therapeutic efficacy of surgical options to reduce the venous diameter by means of extravascular bands, prosthetic sleeves, etc., is uncertain [9, 10], and both carry the risk of serious complications, such as hematoma, incisional infections, and thrombosis [11]. Valve replacement surgery is one of the potential treatments, and the valve sources used for replacement include autologous femoral or axillary veins, allograft venous valves, and prosthetic venous valves [6]. Raju and Taheri et al. treated deep venous valvular insufficiency by transposition or grafting of autologous valve vein segments, but due to the difficulty of the procedure, mismatch of the vessel diameters, and uncertainty of the efficacy of the treatment, this method has not been widely used [12–15]. Besides, fresh or cryopreserved allograft valves are also very limited in clinical application due to the scarcity of donor sources, difficulty in preservation, rejection, and low patency rate [16–19]. In this context, prosthetic venous valves have become a research hotspot for valve replacement therapy, and percutaneous implantation of prosthetic venous valves with good biocompatibility, anti-thrombotic ability, and stability will provide a new therapeutic option for patients with CVD.

Since the first introduction of the concept of the percutaneously implantable prosthetic venous valve, approximately twenty prosthetic valve designs have been documented in the literature [20]; nevertheless, no commercial prosthetic valve product has yet entered the market. In several animal studies of prosthetic venous valves, thrombosis was the leading cause of short-term valve malfunction [21, 22]. This could be related to the physiological properties of the venous system in the lower extremities, as blood flow in the veins is slower, and the vein walls are easily deflated, causing blood stagnation and making thrombosis more likely to form in the venous system. Naturally derived biomaterials are highly biocompatible and cause minimal irritation to the blood system and surrounding tissues; also, patients do not need to take lifetime anticoagulant medication after implantation, which significantly minimizes the risk of bleeding and other adverse events [23]. Some decellularized extracellular matrix (dECM), such as bovine pericardium, porcine heart valves, etc., has been widely used in heart valves and shown satisfactory therapeutic effects. However, the mechanical strength, durability, calcification and especially hemocompatibility of dECM materials are still the most concerning issues [24, 25].

Liu et al. [26] reported glutaraldehyde (GA) crosslinked decellularized fish swim bladder material, which is mostly composed of collagen I, glycosaminoglycans, and abundant elastin. Swim bladder material showed lower immunogenicity and stronger anti-calcification ability than bovine pericardium, as well as stronger mechanical properties and thermodynamic stability [27, 28]. At the same time, swim bladder is derived from aquatic and marine resources, which can effectively circumvent religious restrictions, as well as reduce the risk of disease transmission caused by natural biomaterials of mammalian origin [29, 30]. Prosthetic venous valves for the deep veins of the lower limbs require smaller sizes than the heart valves because of the lower vascular lumen diameter. The thickness of swim bladders can be designed and constructed to 100–200 μm after decellularization treatment, making it easier to meet the demand of valve leaflets for prosthetic venous valves [26, 31]. The purpose of this study was to investigate the hemocompatibility of GA-crosslinked swim bladders, which has not been systematically studied in the venous system. Particularly, hemolysis rate, clotting time, platelet adhesion and activation, and patency in the jugular vein patch model were evaluated and compared to commercially available prosthetic vascular expanded polytetrafluoroethylene (ePTFE) materials, providing a foundation for their future use in the development of venous disease-related developments.

## Materials and methods

### Materials

Fresh swim bladders of silver carp (*Hypophthalmichthys molitrix*) were purchased from a local slaughterhouse (Tianjin, China), and ePTFE material was cut from ePTFE artificial blood vessels (GORE-TEX®, Gore Medical, USA). Activated partial thromboplastin time (APTT), prothrombin time (PT), and thrombin time (TT) assay kits were purchased from Shanghai Sun Biotech Co., Ltd. (Shanghai, China). Phosphate buffer saline (PBS, pH = 7.4) and penicillin-streptomycin solution (100×) were purchased from Wuhan Pricella Biotechnology Co., Ltd. (Wuhan, China). Sodium dodecyl sulfonate (SDS) was purchased from Thermo Fisher (USA). Triton X-100, DNAase, RNAase, and GA were purchased from Sigma (USA). 2.5% (v/v) GA electron microscope fixative, 4’, 6-diamidino-2-phenylindole (DAPI) staining solution, and goat serum blocking solution were purchased from Beijing Solarbio Science & Technology Co., Ltd. (Beijing, China). Alpha smooth muscle actin (α-SMA) polyclonal antibody was purchased from Proteintech Group, Inc. (Wuhan, China). The lactate dehydrogenase (LDH) cytotoxicity test kit was purchased from Beyotime Biotechnology (Shanghai, China). Blood samples were collected from the marginal ear vein of New Zealand white rabbits. New Zealand white rabbits were purchased from Beijing Vital River Laboratory Animal Technology Co., Ltd. (Beijing, China). All animals were kept in the Institute of Radiation Medicine, Chinese Academy of Medical Sciences and were fed with water and food at regular intervals during the follow-up period.

### Preparation of GA cross-linked swim bladder material

Fresh swim bladders were collected, then the surface fat and connective tissue were removed and cleansed with sterile PBS. The swim bladder was submerged in a 1% (w/v) SDS solution and gently shaken at room temperature for 6 h. The swim bladder was cleaned and shaken 3 times with sterile PBS, and a 1% (v/v) TritonX-100 solution was shaken at room temperature for 30 min before being washed with sterile PBS and shaken at 4 °C until the residual reagents were removed. The cleaned swim bladder was immersed in a buffer containing 2 U/mL DNAase and 0.1 mg/mL RNAase and treated with constant temperature oscillation at 37 °C overnight, and the decellularized swim bladder was obtained after being cleaned with sterile PBS 3 times. The decellularized swim bladder was immersed in 0.625% (v/v) GA solution and crosslinked for 12 h at room temperature. Then the GA cross-linked swim bladder was thoroughly washed with PBS and stored in sterile PBS containing 1% (v/v) penicillin-streptomycin at 4 °C.

### Hemolysis test

The GA cross-linked swim bladder and ePTFE artificial blood vessels were cut into 1 × 1 cm rectangular samples and named “Bladder” and “ePTFE”. Both bladder and ePTFE were pretreated in saline at 37 °C for 1 h. Collect venous blood from New Zealand white rabbits using heparin anticoagulation blood collection tubes. The blood samples were centrifuged at 1500 rpm for 10 min. The supernatant was discarded and the same volume of saline was added and gently mixed. Then the mixture was centrifuged again at 1500 rpm for 10 min and the supernatant was discarded. Repeat the procedure 1–2 times until the supernatant became nearly colorless to obtain washed erythrocytes. The washed erythrocytes were diluted into an erythrocyte suspension with saline at a 1:9 (v/v) ratio. The prepared swim bladder and ePTFE were placed into a 1.5 mL ep tube. The negative control, swim bladder, and ePTFE groups were added 100 μL erythrocyte suspension and 900 μL saline, whereas the positive control group was added 100 μL erythrocyte suspension and 900 μL deionized water. Then they were placed at 37 °C for 1 h. After removing the materials, the mixture was centrifuged at 2000 rpm for 10 min, and the supernatant was transferred to a 96-well plate. Each group was assigned 100 μL per well, with three replicate wells. The absorbance was measured at 542 nm. Calculate the hemolysis rate (%) using the following formula:

$${\rm{Hemolysis\; rate}}\left. ( \% \right)=\left(\frac{{A}_{s}-{A}_{0}}{{A}_{1}-{A}_{0}}\right)\times 100 \%$$

*A*_0_ indicates absorbance of the negative control; *A*_1_ indicates absorbance of positive control; *A*_s_ indicates absorbance of sample groups.

### Clotting time measurement

#### APTT

The GA cross-linked swim bladder and ePTFE artificial blood vessels were cut into 1 × 1 cm rectangular pieces, gently rinsed three times in saline, and the water on the surface was absorbed before being placed in 24-well plates (*n* = 3). Venous blood was taken from New Zealand white rabbits in a blood collection tube containing sodium citrate, centrifuged at 1500 × *g* for 15 min, and the supernatant pipetted as platelet poor plasma (PPP). Add 400 μL of PPP to each well to fully submerge the material and incubate at 37 °C for 2 h, 400 μL of PPP was set up a blank control group. For each group, take 100 μL of incubated PPP and mix with 100 μL of pre-warmed APTT test reagent, and then add 0.025 M CaCl₂ solution pre-warmed at 37 °C for 5 min. Immediately stir with a metal wire, the time from the beginning of stirring to the first appearance of fibrin filaments was recorded as APTT, and three parallel samples were measured.

#### PT

PPP preparation and material incubation method were the same as APTT test. 100 μL of incubated PPP was mixed with 200 μL of 37 °C pre-warmed PT assay reagent. Immediately stir with a metal wire and time with a stopwatch. The time from the beginning of the stirring to the first appearance of the fibrin filaments was PT, and three parallel samples were measured.

#### TT

Add buffer to the TT reagent, let it stand for 5–10 min, and shake it gently to dissolve it. If the room temperature is lower than 20 °C, it is necessary to preheat the TT reagent at 25 °C before use. The method of PPP preparation and material incubation is the same as that in APTT test. Take 100 μL of incubated PPP mixed with 100 μL of pretreated TT reagent. Immediately stir with a metal wire and time with a stopwatch. The time from the beginning of the stirring to the first appearance of the fibrin filaments was TT, and three parallel samples were measured.

### Plasma recalcium curve assay

Swim bladder and ePTFE materials were pretreated, and PPP was prepared using the method in APTT test. Pretreated bladder and ePTFE were placed in a 24-well plate, and wells without material were set up as blank controls (*n* = 3). 400 μL of PPP was added to the above wells and incubated at 37 °C for 2 h. After incubation, the samples were removed, and 100 μL of post-incubation PPP was taken from each well to a 96-well plate, and 100 μL of 0.025 M CaCl₂ solution was added to each well. In addition, another 100 μL of post-incubation PPP was taken from each well of the blank control group to a 96-well plate, and 100 μL of PBS solution was added as a negative control. Then the 96-well plate was placed into the multifunctional microplate reader (Thermo Fisher, USA). The plate was shaken for 30 s, and then the absorbance was measured at 405 nm every 30 s for a total of 45 min until the absorbance was basically unchanged.

Lag time and overall coagulation potential were calculated according to the method provided by the study from Alvarez [32]. The plasma recalcium curve is “S” shaped, the absorbance at baseline is A_0_, and the difference between the absorbance and A_0_ after final stabilization is ΔA_405_. Calculate A_c_ = A_0_ + (ΔA_405_ × 0.05), the time corresponding to A_c_ on the curve is lag time (s), which represents the lag time for the initiation of the coagulation cascade reaction. The larger the lag time, the slower the initiation of the coagulation reaction is, which also implies that the influence of the material on the coagulation system is smaller. In addition, the area under the curve of plasma recalcification curve minus the area under the curve of the baseline was calculated as the overall coagulation potential, which reflects the change of the overall ability of blood coagulation after contact with the material.

### Platelet adhesion assay

#### Observation of platelet adhesion

Venous blood was collected from New Zealand white rabbits using blood collection tubes containing sodium citrate, centrifuged at 150 × *g* for 10 min, and supernatants were collected. The platelets were counted using a cell counting plate, and platelet counts were adjusted to 1.0 × 10^8^/mL with saline to make platelet rich plasma (PRP).

GA cross-linked swim bladders and ePTFE artificial blood vessels were cut into 1 × 1 cm rectangular samples and gently rinsed 3 times in saline. Dry the surface with absorbent paper and place them in 24-well plates (*n* = 3). 400 μL PRP was added to each well to completely submerge the samples and incubated at 37 °C for 2 h. The samples were removed, and the unabsorbed platelets were washed away by gentle rinsing in PBS solution three times. The samples were immersed in 2.5% (v/v) GA solution overnight at 4 °C, washed once in PBS, and dehydrated sequentially in a gradient of 50%, 70%, 80%, 90%, and 100% (v/v) ethanol. Then the samples were dried at room temperature before being photographed by scanning electron microscope (SEM, Zeiss, Germany). The number of platelet adhesions and morphological changes on the surface were observed. Ten areas were randomly selected for counting the number of adherent platelets.

#### Semi-quantification of platelet adhesion

Sample pretreatment and PRP preparation were performed as in 2.6.1. Pretreated samples were placed in 24-well plates (*n* = 3). Add 400 μL of PRP to each well to completely submerge the samples, and incubate at 37 °C for 2 h. Gently rinse the samples with PBS solution 3 times to wash away the unabsorbed platelets. Add 300 μL of LDH releasing agent, and incubate at 37 °C for 1 h. Aspirate the liquid after incubation, centrifuge at 400 × *g* for 5 min, and take 120 μL of supernatant in a 96-well plate. Add 60 μL working solution to each well, incubate for 30 min at room temperature with shaking and avoiding light, and measure the absorbance at 490 nm.

### Rabbit jugular vein vascular patch model

#### Jugular vein vascular patch procedure

The swim bladder and ePTFE were cut into 1 × 3 mm rectangles (*n* = 4) and sterilized. ePTFE vascular patches originated from artificial vessels of Gore Medical (GORE-TEX®, USA), and their intimal surface served as the blood-contacting surface when sutured onto the jugular vein; while the smoother visceral surface of the fish bladder was selected as the blood-contacting surface. The rabbits were anesthetized with isoflurane and placed on the operation table. The hair on the neck was shaved, and the skin was cut open. The jugular vein on one side was released, and the proximal and distal ends of the veins were secured with artery clips. A small incision was cut at the blood vessel. The swim bladder or ePTFE was then sutured to the right and left jugular veins, respectively, with 7–0 prolene non-absorbable sutures (Johnson & Johnson, USA). After the repair was completed, the arterial clamps were released to observe the patency of the vessels and whether there was any blood leakage. The skin was sutured, and low-molecular-weight heparin (LMWH) was injected subcutaneously at a dose of 100 IU/kg twice daily after the operation, and the rabbits were observed for any infections, hematomas, and other complications. The rabbits were fed quantitatively with water and food. Ultrasound and venous vessel sampling were performed regularly.

#### Jugular vein ultrasound monitoring and sampling

Ultrasound monitoring of the jugular veins was performed 3 days, 1 week, 2 weeks, 1 month, and 3 months after surgery to detect stenosis, occlusion, and thrombosis of the vessels at the jugular vein patches on both sides, as well as to measure lumen diameters in transverse and blood flow rates. At 1 and 3 months after surgery, the bilateral jugular veins with bladders and ePTFE patches were explanted from rabbits, and the lumens of the removed vessels were gently flushed with heparin-containing saline. The normal conditions of the vessels were observed, and photographs were taken to record whether there was hyperplasia or thrombosis, which were later used for SEM or histological analyses.

#### Surface morphology

The specimen was fixed with 2.5% (v/v) GA overnight at 4 °C, washed once with PBS, and then dehydrated with a gradient of 50%, 70%, 80%, 90%, and 100% (v/v) ethanol. The material was then dried at room temperature and scanned for SEM to determine the number and morphology of haemocytes adhered to the surface, as well as the number of adherent platelets.

#### Smooth muscle cell regeneration

The excised tissues were embedded in OCT (Sakura, Japan) and frozen at −80 °C. Tissues were cut into 6 μm thin slices with a frozen sectioning machine. The OCT on the sections was removed with PBS, and the sections were sealed with goat serum sealing solution for 1 h. The sections were incubated with α-SMA primary antibody overnight at 4 °C and rinsed five times with PBS solution for 5 min each time. Then incubated with secondary antibody for 2 h, washed five times with PBS solution for 5 min each, and sealed with DAPI-containing sealer.

### Data analysis

Immunofluorescence images were measured by ImageJ. Prism 6.0 (GraphPad Software, USA) and Origin 2018 were used for data processing and visualization. A *p* value less than 0.05 was considered statistically significant.

## Results

### In vitro hemocompatibility evaluation

#### In vitro hemolysis experiments

**Fig. 1:**
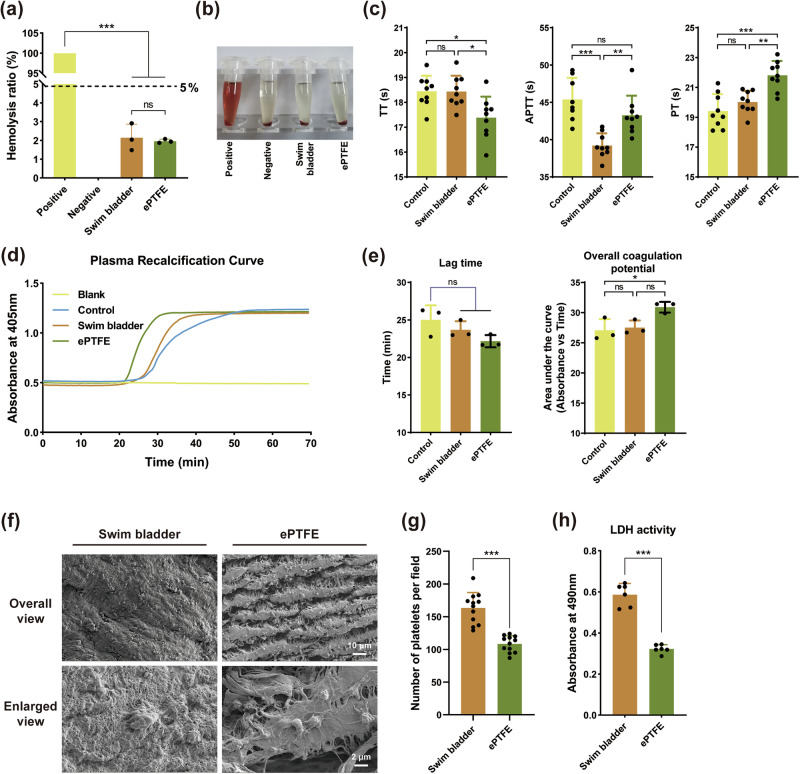
In vitro haemocompatibility evaluation. **a** Hemolysis test (*n* = 3). **b** Images of hemolysis test results in each group. **c** Clotting time test (*n* = 3 independent experiments, with triplicate measurements per sample). **d** Plasma recalcification curve test. **e** Lag time and overall coagulation potential quantitative analysis of plasma recalcification curve (*n* = 3). **f** SEM images of platelet adhesion (*n* = 3 and 4 fields of each sample were selected randomly for data counting), the scale bar is 10 μm and 2 μm, respectively. **g** Platelet adhesion per field counts for SEM images. **h** Semi-quantitative analysis of platelet adhesion by LDH release assay (*n* = 3 and every sample was tested 2 times). “ns” represents no statistical difference. “*” represents *p* < 0.05. “**” represents *p* < 0.01. “***” represents *p* < 0.001

Medical device/material-induced hemolysis is the harmful biological consequence of increased free hemoglobin in plasma produced by red blood cell destruction due to direct surface contact or the release of soluble chemicals. Hemolytic effects of medical devices/materials are often classified into two types: hemolysis caused by substance release from the material and hemolysis caused by mechanical forces on the material’s surface. Material-mediated hemolysis tests include direct contact and leaching methods. In vitro hemolysis experiments using the direct contact approach according to ISO 10993 revealed that the bladder group (2.15 ± 0.72%) and the ePTFE group (1.96 ± 0.11%) had hemolysis rates of less than 5% (Fig. 1a, b). There was no significant difference between the two groups, indicating that the bladder materials would not have significant destructive effects on red blood cells due to their physical properties and chemical substances, which satisfies the basic requirements for use in the cardiovascular system.

#### Clotting time and plasma recalcium curve

The coagulation cascade response has three components: the endogenous pathway, the exogenous pathway, and the common pathway. The endogenous pathway means that all of the coagulation factors involved are derived from the blood and are normally activated by negatively charged foreign surfaces (glass, white vitrified clay, collagen, etc.). The exogenous pathway is initiated by exposure of tissue factors from outside the blood (factor III). And the common pathway means that the endogenous and exogenous pathways combine to activate factor X, resulting in the formation of the prothrombinase complex by Xa, Va, and Ca^2+^ (factor II). The prothrombinase complex enters the common coagulation pathway and activates thrombin, which then cleaves fibrinogen to form fibrin (factor I) and, eventually, a stable thrombus [33].

Coagulation times (APTT, PT, TT) of PPP were measured after contacting with the material (Fig. 1c). APTT represents the time of endogenous coagulation and the common coagulation pathway. Bladder group (39.21 ± 1.64 s) < ePTFE group (43.26 ± 2.63 s) < control group (45.40 ± 2.89 s), indicating that both Bladder and ePTFE can faintly activate the endogenous coagulation pathway, with Bladder activation being more obvious. The PT represents the time of exogenous and common coagulation pathways. The results showed that the control group (19.41 ± 1.14 s) < bladder group (20.02 ± 0.74 s) < ePTFE group (21.8 ± 0.96 s), indicating that both bladder and ePTFE had an inhibitory impact on exogenous coagulation. TT represents the common coagulation pathway. The bladder group (18.43 ± 0.63 s) and the control group (18.45 ± 0.62 s) had a longer thrombin time compared to the ePTFE group (17.28 ± 0.84 s), indicating that the common coagulation pathway was not affected by the Bladder, but was more likely to be activated by ePTFE.

Plasma recalcification time is the reintroduction of calcium into calcium-free plasma to reproduce the coagulation process. In the plasma recalcification assay (Fig. 1d, e), the bladder group (23.69 ± 1.13 s) had a slightly longer time to initiate coagulation compared to the ePTFE group (22.16 ± 0.80 s) without significant difference, and both were lower than the control group (25.01 ± 1.94 s). The overall clotting potential of plasma after contact with the material was compared by calculating the area under the curve of the plasma recalcification curve. The results showed no significant change in the bladder group compared to the control group, indicating the unactivated coagulation potential, whereas there was an increase in coagulation potential in the ePTFE group compared to the control group. Overall, the results of the plasma recalcification assay show that the swim bladder does not significantly activate the complete coagulation system, whereas ePTFE does.

#### Platelet adhesion

Platelet adhesion is the initial response engaged in normal haemostasis following vascular injury. Platelets cling to one another and aggregate, triggering a cascade of coagulation processes that result in the creation of a thrombus that blocks the blood vessel. Electron microscopy revealed a larger number of platelets in the bladder (163.40 ± 23.60 per field) than in ePTFE (108.50 ± 12.38 per field) (Fig. 1g). In addition, the semi-quantitative platelet adhesion assay by LDH revealed that the OD value of bladder (0.58 ± 0.05) was higher than that of ePTFE (0.32 ± 0.02) (Fig. 1h), confirming more platelet adhesion. The semi-quantitative results were similar to the quantitative data from electron microscopy. In vitro experiments, platelets exhibited more adhesion to the surface of the swim bladder, which may be related to the higher surface roughness of the swim bladder [34].

### In vivo hemocompatibility evaluation

#### Vascular diameter and flow rate measurement

**Fig. 2:**
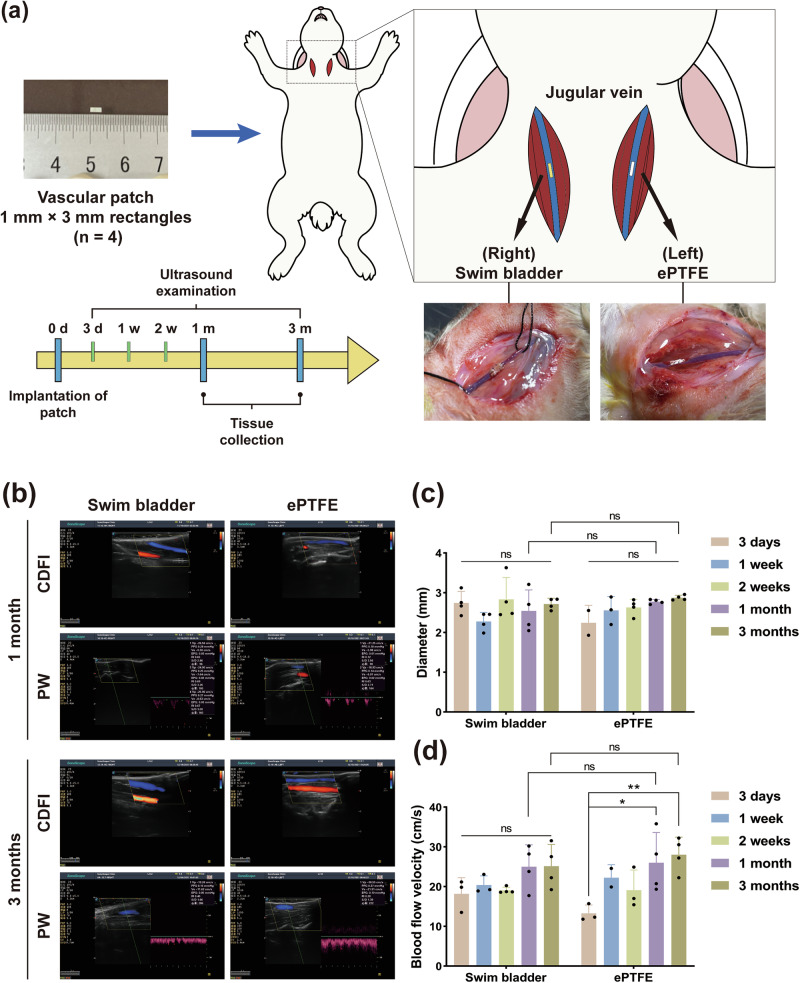
In vivo hemocompatibility evaluation and ultrasound follow-up. **a** Schematic diagram of the rabbit jugular vein vascular patch model and follow-up time flow (*n* = 4). **b** Ultrasound longitudinal cross-section for vascular patency and blood flow velocity measurement. CDFI color Doppler flow imaging, PW pulsed wave. **c** Vessel diameter measurements at the patch at 3 days, 1 week, 2 weeks, 1 month, and 3 months postoperatively. **d** Blood flow velocity measurement at the patch at 3 days, 1 week, 2 weeks, 1 month, and 3 months postoperatively. “ns” represents no statistical difference. “*” represents *p* < 0.05. “**” represents *p* < 0.01

The swim bladder and ePTFE were cut into 1 × 3 mm rectangles and tested in the rabbit jugular vein vascular patch model (Fig. 2a). Postoperative monitoring of the rabbit jugular vein with ultrasound imaging equipment revealed that the vessel’s wall was clearly visible in the transverse section, and the lumen was compressible and very resilient. The lumen of the longitudinal interface was permeable without hypoechoic, and the lumen could be compressible and unobstructed. The ultrasound results showed that the vessels repaired by both groups of materials were smooth and free of obstruction and stenosis at 1 month and 3 months (Fig. 2b). The vessel diameters remained stable during the 1-month period, with no significant difference between the bladder group (2.61 ± 0.49 mm) and the ePTFE group (2.60 ± 0.28 mm). At 3 months, there was also no significant difference between the bladder group (2.72 ± 0.14 mm) and the ePTFE group (2.80 ± 0.10 mm) (Fig. 2c). Maximum blood flow rates were lower in the bladder group (25.00 ± 5.52 cm/s for 1 month and 25.15 ± 5.47 cm/s for 3 months) than the ePTFE group (26.03 ± 7.59 cm/s and 28.06 ± 4.40 cm/s), but with no significant difference (Fig. 2d). Further, vessels fixed by bladder could maintain the maximum blood flow rates steady from 1 month to 3 months, indicating a mature and stable state.

#### Explanted venous vessels and patency rates

**Fig. 3:**
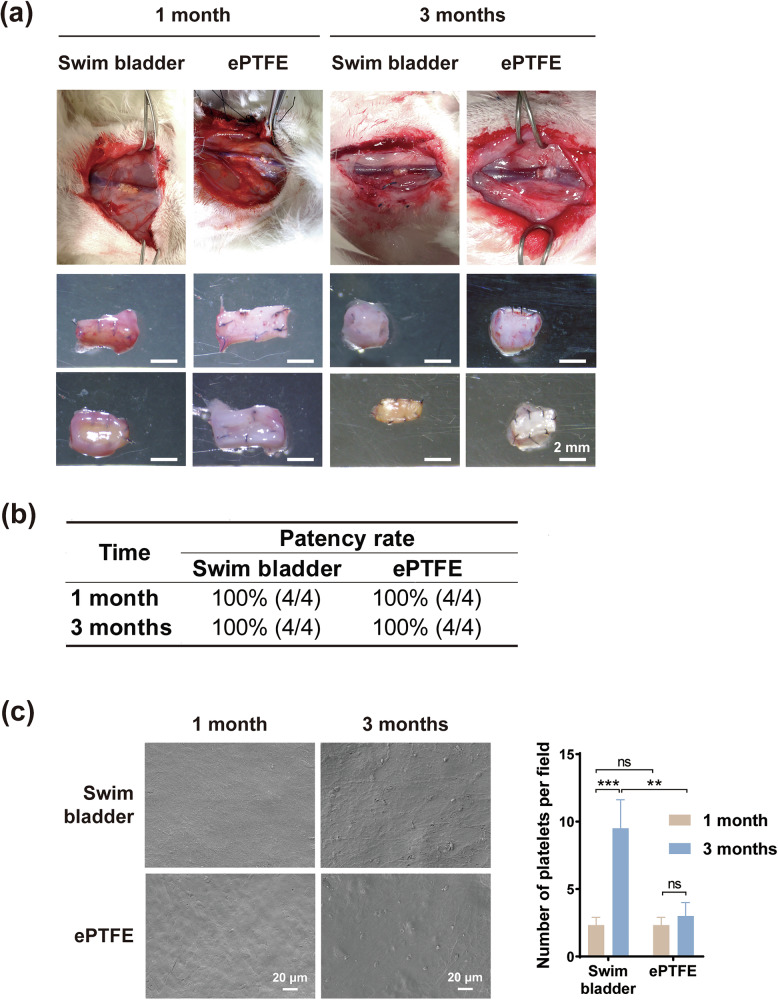
Explanted venous vessels and electron microscopy for in vivo hemocompatibility evaluation. **a** Venous vessels explanted at 1 and 3 months postoperatively. **b** Vascular patency statistics at 1 and 3 months postoperatively. **c** SEM images of vascular specimens and statistics of platelet adhesion number at 1 and 3 months postoperatively, scale bar is 20 μm. “ns” represents no statistical difference. “**” represents *p* < 0.01. “***” represents *p* < 0.001

Surgery was performed under a body microscope, and both jugular veins were repaired with no blood loss. At 1 month and 3 months, the venous vessels fixed by the bladder patches were patent (Fig. 3b), with no thrombus, stenosis, or hyperplasia. The outer/inner wall of the lumens were clean and smooth at both time points, and the animal studies indicated that the bladder patches were very compatible with venous blood hemoglobules, which was in accordance with the in vitro data (Fig. 1a, b).

#### Platelet adhesion on the explanted vascular patch

The SEM images show that the swim bladder and ePTFE patch were smooth and intact at 1 month after implantation, with little adherent platelet (2.33 ± 0.47 per field of view) and no aggregation; 3 months after implantation, there were some platelet adhesion and aggregation on the surface of the swim bladder vascular patch, with 9.50 ± 1.50 adherent platelets per field of view, slightly more than the ePTFE (3.00 ± 0.82 per field of view) (Fig. 3c).

#### Immunofluorescence staining

**Fig. 4:**
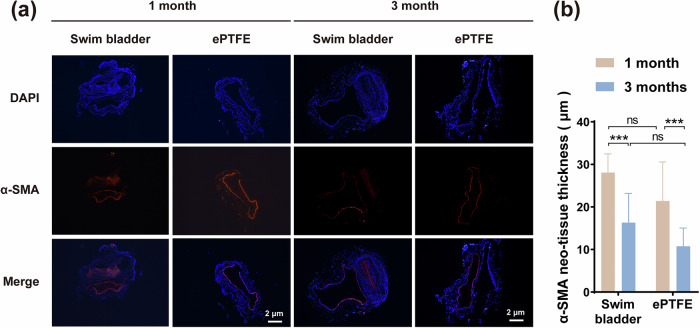
α-SMA immunofluorescence staining inside the venous vessel lumen **a** α-SMA immunofluorescence staining at 1 and 3 months postoperatively. Scale bar is 2 μm. **b** Thickness of the smooth muscle layer inside the venous vessel lumen calculated from the immunofluorescence staining, “ns” represents no statistical difference. “***” represents *p* < 0.001

α-SMA immunofluorescence staining was conducted to evaluate the smooth muscle layer formed inside the venous vessel lumen (Fig. 4a). Results showed that the jugular venous vessels repaired with both bladder and ePTFE materials showed an α-SMA positive phenotype at 1 and 3 months postoperatively, indicating that both materials generated a thin layer of smooth muscle cells during the repair process, and the smooth muscle tissue was uniform and continuous. At 1 month, the thickness of the smooth muscle layer inside the venous vessel lumen repaired with bladder was 28.07 ± 4.21 μm, which was thicker than that of the ePTFE group (21.4 ± 8.70 μm). At 3 months, the thickness of the smooth muscle of the venous vessels repaired with both the two materials decreased slightly, which was 16.30 ± 6.64 μm for the bladder group and 10.74 ± 4.15 μm for the ePTFE group. Based on the results of smooth muscle fluorescence staining, it can be inferred that the matrix of fish swim bladders is more suitable for tissue cell growth and infiltration. In addition, we hypothesized that there would be thickening of the smooth muscle during the vessel repairs. With increasing implantation time, the thickness of the smooth muscle layer was reversed, gradually getting thinner and stabilized [35].

## Discussion

In our previous research, we discovered that the swim bladder was made up of elastin, type I collagen, and glycosaminoglycans, with elastin being especially plentiful. The GA-crosslinked swim bladder has an ultimate tensile strength of 7.7 ± 0.8 MPa and an axial modulus of elasticity of 19.2 ± 5.8 MPa. After 12 days of digestion, GA-crosslinked swim bladders showed a weight loss rate of 5.8 ± 1.8% for collagenase and 22.6 ± 3.2% for elastase. The mechanical properties of ePTFE are strongly dependent on its microstructure (the morphology of nodes and fibers), which in turn is determined by raw materials and processing conditions such as draw ratio, draw rate, and temperature [36]. Commercial vascular grafts generally exhibit tensile strengths within the 20–50 MPa range. The elongation at break of ePTFE can reach 100–400%, demonstrating excellent flexibility and tear resistance. The cell viability of the three cell lines, L929, HUVECs, and RIVCs, was greater than 80% after 24 h of incubation with the extract, which is consistent with the qualifying standard in ISO 10993-10 Biological Evaluation of Medical Devices [26]. It demonstrates that swim bladders have the potential to become a new cardiovascular biomaterial due to their excellent mechanical qualities, anti-enzymatic stability, and cytocompatibility. However, there is no comprehensive evaluation experiment on the hemocompatibility of swim bladders. Thus, in this study, the blood compatibility of the swim bladder is evaluated using ISO 10993-4 to ensure its safety as an implanted device. The swim bladder material demonstrated good blood compatibility in the venous system. It was equivalent to commercially available artificial venous vessel material ePTFE in terms of hemolysis rate, clotting time, and blood platelet adhesion and even showed unique advantages in some aspects. In a 3-month follow-up of the rabbit jugular vein vascular patch model, the swim bladder-derived blood vessels at the vascular patch remained patent, and the vascular flow rate was between 25.00 ± 5.52 cm/s and 25.15 ± 5.47 cm/s during 3 months postoperatively, which is normal (Fig. 2b–d). There was no obvious thrombus formation during the whole testing period until the material was explanted, and a homogeneous smooth muscle layer gradually formed as the implantation time increased (Fig. 4). This study illustrates the potential of swim bladder material as the vascular patches or venous valves in treating CVD.

The low flow rate, high tissue metabolite concentration, and thin-walled structure of the venous system place strict demands on biomaterials’ antithrombotic and compliance capabilities. The thickness of fresh swim bladder material is about 100–300 μm, and after decellularization and cross-linking, the thickness can be controlled at 200–400 μm [26], the thickness of which is highly compatible with the physiological thickness of natural venous valve leaflets (about 200 μm). This structural adaptability was significantly better than that of traditional valvular materials such as bovine pericardium (400–600 μm), which provides an advantage for its application in endovascular catheter delivery and minimally invasive surgery [26, 37]. Furthermore, the match between the mechanical properties of the material and the vessel wall at the implantation site has a significant impact on long-term patency [38]. Swim bladders’ high elastin content may confer flexibility that is similar to the vein wall [26]; while ePTFE may result in increased local stress or blood flow disturbances due to the mismatch of the local mechanical properties and is prone to excessive proliferation of smooth muscle cells after prolonged implantation, which may increase the risk of restenosis and thrombosis [39].

In in vitro experiments, the swim bladder material exhibited higher platelet adhesion (Fig. 1f). This may be related to the higher surface roughness of the swim bladder. But in vivo experiments did not observe much platelet adhesion or any thrombosis (Fig. 3), which is more persuasive. On the one hand, this may be attributed to the dynamic regulation of the in vivo microenvironment, which can better simulate actual application scenarios. On the other hand, glycosaminoglycans (e.g., chondroitin sulfate) retained on the decellularized surface of the swim bladder can inhibit complement activation and inflammatory response [40]. More importantly, the hydrophilic property of the swim bladder surface facilitates the preferential adsorption of plasma proteins less relevant to thrombosis, such as albumin, and facilitates the formation of an intact and contiguous endothelium, which can cover platelet adherence sites and reduce the risk of thrombosis [41]. This may be one of the important factors why swim bladders show better antithrombotic properties in long-term in vivo implants than in in vitro experiments. It is noteworthy that a small amount of platelet adhesion still existed on the surface of the swim bladder at 3 months (Fig. 3c), which may be related to its surface microtopography. In future study, optimization of the surface microstructure by surface anticoagulant coating modification (e.g., heparin coating) or nano-coating technology may further improve the antithrombotic properties of the swim bladder [42]. The current study found that 1 month after swim bladder patch implantation in the rabbit jugular vein, the gradual decrease in the thickness of the smooth muscle layer indicated that the material’s integration with the host tissue was homeostatic (Fig. 4). The α-SMA immunofluorescence staining results show that smooth muscle cells grow better in the swim bladder. The decrease in tissue thickness at 3 months compared to 1 month could be explained by the transition to a quiescent/steady state of vascular healing.

The durability of natural biomaterials is a major issue that limits their long-term use. After decellularization, swim bladder reserves their natural elastin cross-linking network, and GA cross-linking can improve the material’s mechanical stability by stabilizing the collagen structure and forming stable chemical bonds between adjacent proteins in the decellularized matrix [43]. However, the chemical residues formed following GA cross-linking are cytotoxic and may cause persistent inflammation and calcification. Future studies can assess the local inflammatory response and blood levels of inflammatory markers (e.g., TNF-α, IL-6) following long-term implantation to validate their biosafety. In future studies, we can also adopt improved crosslinking processes of natural materials, such as supercritical CO₂ decellularization treatment [44] and composite crosslinking strategies with dual crosslinking agents [45] in relation to the various needs of clinical applications. These crosslinking processes reduce the potential toxicity caused by the preparation process and efficiently remove immunogenic substances in the substance while retaining the extracellular matrix components to the greatest extent possible. It can also improve the mechanical stability and the long-term anti-calcification ability of the material.

Finally, the results of in vitro and in vivo hemocompatibility study of fish swim bladder-derived biomaterials demonstrated that they are biocompatible and do not stimulate the coagulation system or blood cells significantly for the application of venous vascular patch. The danger of thrombosis was low, and swim bladders may be employed as vascular patches in the venous system or prosthetic venous valve leaflet materials. Based on the unique properties of swim bladder materials, bionic multilayer structural composite scaffolds made of swim bladders and synthetic polymer materials can be built using 3D printing technology in the future, balancing mechanical strength and bioactivity for complementary performance. Furthermore, surface functionalization (e.g., heparin coating or RGD peptide modification) can further restrict platelet adherence, encourage endothelial cell to migration directionally and produce fast reendothelialization, which can also boost antithrombotic performance.

## Limitation

For the blood contact safety in the application of CVD, this study focuses on the assessment of hemocompatibility of fish swim bladders in vivo and in vitro, but there are still some limitations. First, there seems to be some conflict in the data we obtained in the clotting time assay and plasma recalcification assay. The shortened APTT values of swim bladders relative to the control group suggest a possible promoting effect on endogenous coagulation. However, in the plasma recalcium assay, the coagulation initiation time of the swim bladder was not statistically different from that of the control. Comparing the experimental principles of the two, the APTT was measured with the addition of substances that stimulate the activation of coagulation, while the plasma recalcium assay simply added calcium ions to restore the ability of the coagulation system to work properly, which in turn provides a more realistic reflection of the effect of the material on the coagulation system after exposure to blood. Taking the above analyses into account, we believe that the results of the plasma calcium replication test are more credible, but additional experiments are needed in the future to further verify our speculation. Second, as the leaflet material of the prosthetic venous valve or venous vascular patch, the hydromechanical property is one of the key evaluation indexes, but this has not been systematically determined in this paper. Using computer fluid dynamics to simulate local hemodynamics and vessel wall stress after implantation, as well as analyzing the hemodynamic adaptability of swim bladder materials in different venous segments (e.g., femoral vein vs. popliteal vein), could provide effective methods to quickly evaluate material function. Furthermore, the hemodynamics of the jugular vein of New Zealand white rabbits differed significantly from the deep veins of the lower limbs in humans. The human venous system is subjected to higher hydrostatic pressure and long-term contraction of the lower limb muscle pump, as well as changes in body position [46]. The pressure on the lower limb venous vessels and venous valves to undergo dynamic changes for an extended period, potentially imposing more demanding requirements on the mechanical durability of the implanted materials. In the previous study, we have already confirmed the durability of swim bladder as valve leaflets in the transcatheter aortic valve [47]. As a result, it is required to investigate the fatigue properties of swim bladder materials in dynamic mechanical environments, as well as validate their mechanical durability in large animal models (e.g., canines or primates) capable of simulating the long-term mechanical loading of the human venous system. Overall, its possibility for clinical translation requires more investigation across numerous dimensions, including biological mechanisms, in situ implantation model performance and long-term hemodynamics performance. In addition, reducing LMWH dosage and employing end-to-end anastomosis models is beneficial for amplifying potential differences between swim bladder and ePTFE.

## Conclusion

For the application of CVD, in this study, the blood compatibility of the swim bladder is evaluated using ISO 10993-4 “Biological Evaluation of Medical Devices-Part 4” to ensure its blood contact safety as an implanted device. The effects of the materials on endogenous and exogenous coagulation were investigated using coagulation time and plasma recalcification studies. Both swim bladder and control group ePTFE can activate the endogenous coagulation pathway, and both materials inhibit exogenous coagulation. Meanwhile, in vivo experiments were conducted to further study the swim bladder material, and the rabbit’s jugular vein was repaired with swim bladder and ePTFE patches. The swim bladder exhibited better compliance than ePTFE, and there was no leakage when the suture was done. The survival rate of rabbits during the in vivo studies was 100% at 1 and 3 months, and the repaired vessel had a 100% patency rate after fixing by swim bladder patches on the jugular vein.

In conclusion, the swim bladder has great blood compatibility, and its durability can be tested under dynamic and high-load situations in the future. Its antithrombotic performance can also be improved by modifying the sample preparation and surface modification process. Swim bladders are likely to be used in the development of venous system-related devices, resulting in safer and more effective treatments for CVD patients.
